# Pseudohypoparathyroidism type I‐b with neurological involvement is associated with a homozygous PTH1R mutation

**DOI:** 10.1111/gbb.12308

**Published:** 2016-08-24

**Authors:** R. Guerreiro, J. Brás, S. Batista, P. Pires, M. H. Ribeiro, M. R. Almeida, C. Oliveira, J. Hardy, I. Santana

**Affiliations:** ^1^ Department of Molecular NeuroscienceUCL Institute of NeurologyLondonUK; ^2^ Department of Medical Sciences, Institute of Biomedicine – iBiMEDUniversity of AveiroAveiroPortugal; ^3^ Neurology DepartmentCentro Hospitalar e Universitário de CoimbraCoimbraPortugal; ^4^ Hospital do Santo EspíritoTerceiraPortugal; ^5^ CNC – Center for Neuroscience and Cell Biology; ^6^ Faculty of MedicineUniversity of CoimbraCoimbraPortugal

**Keywords:** Dementia, exome sequencing, Pseudohypoparathyroidism Type I‐b, PTH1R

## Abstract

Pseudohypoparathyroidism type 1b (PHP1b) is characterized by hypocalcemia, hyperphosphatemia, increased levels of circulating parathyroid hormone (PTH), and no skeletal or developmental abnormalities. The goal of this study was to perform a full characterization of a familial case of PHP1b with neurological involvement and to identify the genetic cause of disease. The initial laboratory profile of the proband showed severe hypocalcemia, hyperphosphatemia and normal levels of PTH, which was considered to be compatible with primary hypoparathyroidism. With disease progression the patient developed cognitive disturbance, PTH levels were found to be slightly elevated and a picture of PTH resistance syndrome seemed more probable. The diagnosis of PHP1b was established after the study of family members and blunted urinary cAMP results were obtained in a PTH stimulation test. Integration of whole genome genotyping and exome sequencing data supported this diagnosis by revealing a novel homozygous missense mutation in PTH1R (p.Arg186His) completely segregating with the disease. Here, we demonstrate segregation of a novel mutation in PTH1R with a phenotype of PHP1b presenting with neurological symptoms, but no bone defects. This case represents the extreme end of the spectrum of cognitive impairment in PTH dysfunction and defines a possible novel form of PHP1b resulting from the impaired interaction between PTH and PTH1R.

Hypoparathyroidism (HP) and pseudohypoparathyroidism (PHP) are a group of heterogeneous conditions in which hypocalcemia and hyperphosphatemia occur as a result of deficient parathyroid hormone (PTH) secretion or end‐organ PTH resistance (Thakker 2004). As a consequence of hypocalcemia, patients typically present with signs and symptoms of peripheral neuromuscular hyperexcitability (perioral numbness, paresthesias of the distal extremities or muscle cramping) that can progress to carpopedal spasm or tetany. Chronic HP and, more rarely, PHP are associated with central neurologic manifestations like epileptic seizures and extrapyramidal signs (Bhadada *et al.* 2011; Guberman & Jaworski 1979; Mitchell *et al.* 2012). The classical radiological finding is calcification of the basal ganglia (Faissolle *et al.* 2008; Goswami *et al.* 2012; Kahloul *et al.* 2009) and encephalopathy (Gupta *et al.* 2011; Handa *et al.* 1995; Oechsner *et al.* 1996) or rapidly progressive dementia (Adorni *et al.* 2005; Mateo & Gimenez‐Roldan 1982; Nicolai & Lazzarino 1994; Zambrana Garcia *et al.* 1998) have been described, but are usually responsive to treatment and rarely progressive (Stuerenburg *et al.* 1996).

Hypoparathyroidism is mainly an acquired disease, characterized by low or inappropriately normal levels of PTH, resulting from damage or surgical excision of the parathyroid glands or autoimmune mechanisms. Genetic forms of HP are rare and usually associated with mutations in four genes: calcium‐sensing receptor (*CASR*) (Bai *et al.* 1996; Pearce *et al.* 1996; Watanabe *et al.* 1998), glial cells missing homolog 2 (*GCM2*) (Ding *et al.* 2001), guanine nucleotide‐binding protein alpha‐11 (*GNA11*) (Mannstadt *et al.* 2013), and *PTH* itself (Arnold *et al.* 1990; Parkinson & Thakker 1992; Sunthornthepvarakul *et al.* 1999).

Pseudohypoparathyroidism was first described by Fuller Albright *et al.* (1942) (Albright F., 1942) and the nomenclature used was based on the observation of renal resistance to PTH and increased levels of the hormone. Pseudohypoparathyroidism is divided into types I and II according to the absence or presence, respectively, of an increment in urinary cAMP excretion in response to exogenous PTH administration. PHP type 1 is further subdivided into PHP1a, PHP1b and PHP1c. PHP1a, Albright's hereditary osteodystrophy (AHO), is associated with multiple hormone resistance, including thyroid stimulating hormone (TSH) and gonadotropins, causing hypothyroidism and gonadal failure, respectively. The disease is explained by a decrease in the levels and activity of G protein. PHP1c also exhibits AHO, but the molecular mechanism seems to be related with a defect in the catalytic unit of adenylate cyclase or *GNAS* mutations leading to defective Gs‐alpha‐receptor interactions and consequent hormone resistance (Thiele *et al.* 2011). In PHP1b the observed hormone resistance is mostly limited to PTH, patients do not exhibit AHO and have normal G protein activity. In theory, these typical findings in PHP1b could potentially be caused by a defect in the type 1 PTH receptor PTH1R (OMIM 168468), however, sequencing of the gene in PHP1b patients found no mutations in protein‐coding exons or gene promoter regions (Jan de Beur *et al.* 2000; Schipani *et al.* 1995b), and no linkage has been shown so far to the *PTH1R* locus in PHP1b families (Fukumoto *et al.* 1996, 1998). Given the complexities in the molecular, biochemical and physical features of PTH disorders, molecular testing is critical for achieving a clear diagnosis and validating the inheritance pattern in any given family. Nonetheless, the majority of familial cases of isolated HP and PHP remain unexplained most likely because adequate evaluation of known genes has not been undertaken, and because other genes must be involved in the molecular pathogenesis. Obvious potential candidate genes are the PTH receptor genes, nevertheless, to our knowledge; no mutations in these genes have been previously identified as causing these disorders. Here we report a Portuguese family with PHP type 1b with heterogeneous PTH levels and neurological manifestations, due to a novel *PTH1R* mutation.

## Methods

### Genetic analyses

**Figure 1:**
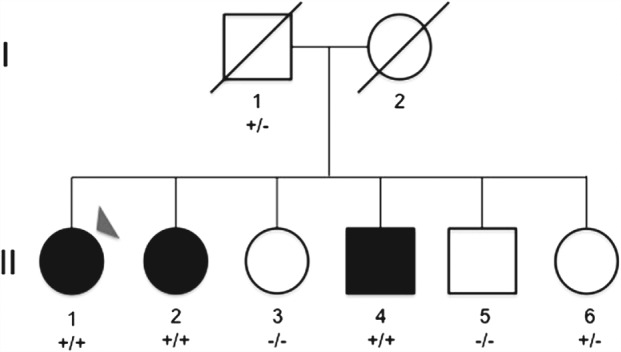
**Pedigree of the studied family.** Black symbols represent affected and white symbols represent unaffected family members. DNA was available from all siblings (generation II) and from the father (I.1). An arrowhead represents the index case. Results for segregation of the mutation with disease are shown below each symbol with all unaffected individuals carrying either 1 (+/−) or 2 (−/−) reference alleles and all affected siblings harbouring the mutation in the homozygous state (+/+). The ‘+’ symbol represents presence of the mutation and the ‘–’ symbol represents absence of the mutation.

All studied individuals gave written informed consent for this study, which was approved by the University Hospital of Coimbra ethics committee and complies with the Declaration of Helsinki. In order to assess the presence of large structural variants and to perform homozygosity mapping, DNA samples from six siblings (II.1–II.6, Fig. 1) were run on Illumina's HumanOmniExpress BeadChips as per manufacturer's instructions (Illumina, San Diego, CA, USA). Data was visualized and analysed using the GenomeStudio Data Analysis Software (Illumina). After exclusion of molecular changes in *GNAS* by direct sequencing, Multiplex Ligation‐dependent Probe Amplification (MLPA) and methylation tests in the index case, exome sequencing was performed in three siblings (II.1, II.2 and II.3, Fig. 1) with Illumina'sTruSeqExome Enrichment according to the manufacturer's instructions. Sequencing was performed in Illumina's HiSeq2000 using 100 bp paired‐end reads. Sanger sequencing was used to establish segregation of the mutation with disease status in all samples available for testing. Details for these analyses are given in the Supporting Information.

### PTH stimulation test

A PTH stimulation test was performed in the index case using subcutaneous recombinant human PTH‐(1–34). In accordance with a protocol recently described (Todorova‐Koteva *et al.* 2012), urinary cAMP and urinary phosphate were measured before PTH injection (baseline) and 2 and 4 h after the PTH challenge.

## Results

### Clinical and laboratory findings in the index case

The index case is a Caucasian 68 years‐old female, with the diagnosis of epilepsy at age 22, controlled with carbamazepine 800 mg/day. She had normal psychomotor development, no history of learning problems, and completed 6 years of education. Her past medical history was relevant for bilateral cataract with surgical extraction at the age 40, as well as a femur fracture at 44 years.

**Figure 2:**
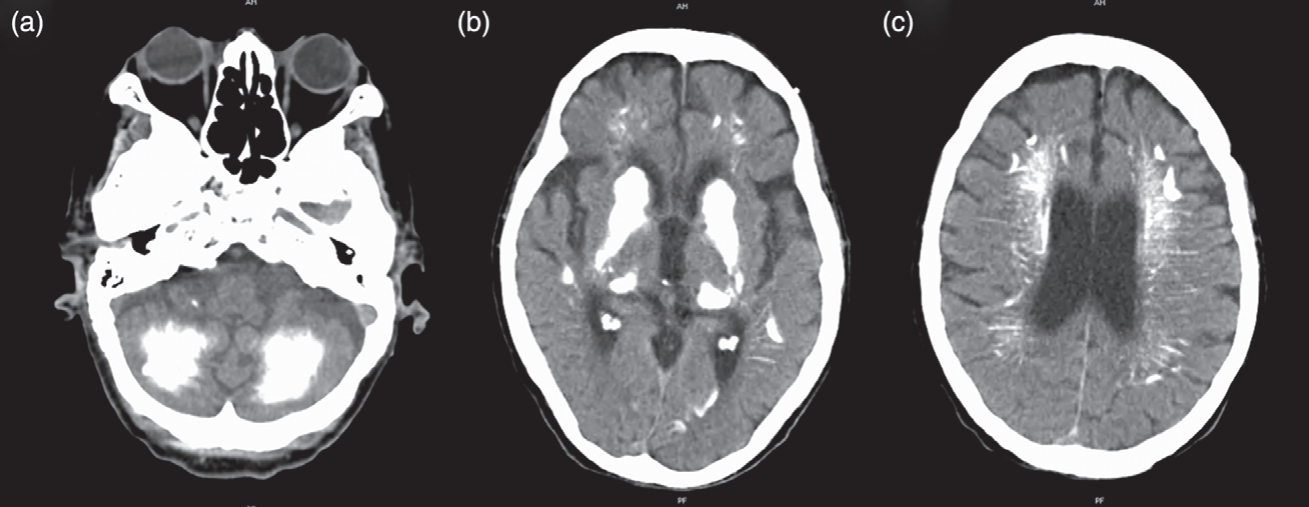
**Computed tomographic scan of the patient's brain.** CT scans show extensive, bilateral calcification of dentate nuclei and cerebellar white matter (a), basal ganglia (b) and centrum semiovale and periventricular white matter (c).

At 49 years old, complex partial seizures became recurrent and refractory to medication and she was first admitted to our inpatient unit for further investigation. Symptoms suggestive of tetany (mainly spasms of the inferior limbs) were present. On general physical examination she had no apparent dysmorphic features, brachidactyly, other deformities or subcutaneous calcifications. The bedside cognitive evaluation was unremarkable. Neurological examination revealed a slightly spastic ataxic gait, without cognitive impairment or other central nervous system (CNS) signs. In the diagnostic investigation, brain computed tomography (CT) scan showed multiple calcifications of basal ganglia and cerebellum (Fig. 2). Electrocardiogram (ECG) was normal, and electroencephalogram (EEG) revealed a generalized slowing and bilateral temporal paroxysmal activity, predominantly on the left side. Serum biochemical analysis presented severe hypocalcemia and hyperphosphatemia with normal proteins and renal function (Table 1). Parathyroid hormone levels were found to be within the normal range on successive evaluations. Thyroid function was normal and there were no other endocrine defects. The patient was at this time diagnosed with primary HP and started oral calcium supplementation with calcium carbonate 4 g/day and calcitriol 0.25 µg/day, in addition to maintenance of carbamazepine. Thereafter her physical condition normalized, seizures stopped and soon after hospital discharge she recovered her normal life and professional activity. Meanwhile, she was regularly followed at the endocrinology outpatient department, with dose adjustments of the calcium carbonate and calcitriol in accordance with serum calcium and phosphorus levels. In November 2009, at 66 years old, she developed an acute confusional state following an orthopaedic surgery, without complete recovery after discharge. Afterwards, the family noticed a progressive cognitive, although fluctuant, deterioration and functional decline in daily living activities. She was admitted to our inpatient department three months after the beginning of these symptoms. The neurological examination revealed moderate to severe cognitive deterioration (see Supporting Information for details). At this time, laboratory analyses revealed a mild hyperphosphatemia with normocalcaemia and a slight elevation of PTH levels (Table 1). Based on the blunt interval change in the urinary cAMP over time (Table 2), the patient was found to have resistance to PTH action and the diagnosis of PHP was assumed. Since mutations of *GNAS* are known to be associated with PTH resistance, the gene was tested at this time with negative results. Even though pharmacological intervention was tried, namely with in‐care rigorous control and maintenance of normal serum calcium and phosphorus levels, through the careful adjustment of the calcium carbonate and calcitriol supplementation, the patient became bedridden, with hypokinetic rigidity, mutism and sporadic motor agitation and died after 2 years. Progression of clinical features, EEG and biochemical profile over time are summarized in Table 1.

**Table 1:** Clinical progression and correlation with calcaemia, phosphataemia, parathyroid hormone levels and electroencephalogram findings in the index case

	December 1992 (49 yo)	1993–2010	February 2010 (66 yo)	August 2011 (67 yo)	November 2011 (68 yo)	September 2012 (68 yo)
Clinical manifestations	PC seizures; Tetania	None	Confusional syndrome; Progressive cognitive deterioration	Recovery of motor and mental functions	Cognitive and motor worsening	Bedridden, with hypokinetic rigidity and mutism
MMSE	30/30		13/30	25/30	0/30 (Mutism)	0/30 (Mutism)
Calcemia (mg/dl) (Normal range: 8.1–10.4)	3.9	7–8	8.4	8.3	9.1	8.5
Phosphatemia (mg/dl) (Normal range: 3.0–5.0)	6.7	4.5–5.5	5.2	4.6 mg/dl	3.8	4.0
PTH (pg/ml) (Normal range: 9–72)	45	35–45	74	13.2	26	36
EEG	Generalized slowing; bilateral temporal spikes		FIRDA; bilateral temporal spikes			Paroxysmal activity on temporo‐occipital regions

yo, years old (age of the patient in each evaluation); EEG, electroencephalogram; FIRDA, frontal intermittent rhythmic delta activity; PC, Partial Complex; PTH, Parathyroid hormone; Dec, December; Feb, February; Aug, August; Sept, September.

**Table 2:** Results of the parathyroid hormone stimulation test in the index case.

	Baseline	2 h after PTH	4 h after PTH
Urinary cAMP/Cr (nmol/mg)	18.70	13.80	15.50
Urinary PO4 (mmol/L)	3.8	13.5	8.8

cAMP, 3′,5′ cyclic adenosine monophosphate; Cr, creatinine; PO_4_, phosphate; PTH, parathyroid hormone.

### Family history and investigation

A key feature of this case was the possible familial aggregation, pointing to a genetic form of disease. Family history evaluation and investigation revealed that the patient's parents were both from the same small village and a common ancestor was mentioned (Fig. 1). The patient's mother had died of stroke in late life with no history of epilepsy, neurological or metabolic/bone disease. The father was always asymptomatic and refused laboratory or brain imaging, but consented to DNA sampling and analysis. He died 2 years after assessment, at 92 years of age without relevant symptoms. As is indicated in Fig. 1, the second generation included six siblings, 55 to 68 years old, of whom the index patient is the oldest. Subjects II.3, II.5 and II.6 were asymptomatic and with serum Ca, Ph and PTH levels within the normal range (Table 3). Subjects II.2 and II.4 both had the diagnosis of parathyroid dysfunction with cerebral calcification. Patient II.2 at the age of 50 experienced speech problems and, considering the neurological condition of her sister, requested laboratory investigation. Results were remarkable for hypocalcemia, hyperphosphatemia, high levels of PTH, normal thyroid function and cerebral calcifications. The diagnosis of PHP was proposed and the symptoms reverted with calcium carbonate and calcitriol. At the moment, with 67 years, she mentions subjective memory complaints but declined neuropsychological examination. A recent brain CT disclosed extensive calcifications of the basal ganglia, periventricular white matter, centrum semiovale and cerebellar white matter. Recent laboratory tests confirmed the high levels of PTH with normocalcaemia and normophosphataemia (Table 3). Given the family history, her brother (subject II.4, 62 years old), also had a diagnosis of parathyroid dysfunction at the age of 45, although being asymptomatic. Laboratory tests confirmed similar results of hypocalcemia, hyperphosphatemia and high levels of PTH. Treatment with calcium carbonate and calcitriol was prescribed. Recent investigations confirmed very high levels of PTH with normocalcemia/normophosphatemia and the presence of pallidal calcifications on brain CT (Table 3).

**Table 3:** Biochemical and imagiological characteristics of the family members (affected members are under treatment)

	II‐1 (index patient)	II‐2	II‐3	II‐4	II‐5	II‐6
Calcemia (8.4–10.2 mg/dl)	8.5	8.7	9.4	8.6	9.3	8.4
Phosphatemia (2.5–4.5 mg/dl)	4.0	3.6	3.8	3.5	3.1	2.9
PTH (10–70 pg/ml)	35–74	172–198	53.7	365–500	47	31
TSH (0.27–4.20 mUI/L)	1.5	1.8	0.94	2.3	2.1	0.83
Cranial CT scan	Multiple calcifications	Multiple calcifications	N	Pallidal calcifications	N	N
Medication	Calcium carbonate + calcitriol	Calcium carbonate + calcitriol	–	Calcium carbonate + calcitriol	–	–

The identifications for family members relate to Fig. 1. N, normal; ‘‐’, no medication.

### Genetic analyses

Homozygosity mapping in the six siblings revealed three large regions (>1 Mb) of loss of heterozygosity in chromosome 3 that were present in all affected, and absent in unaffected siblings (Table 4 and Fig. S1, Supporting Information).

**Table 4:** Regions of extended homozygosity (>1 Mb) shared by affected and absent in unaffected siblings

Chromosome	Start (bp)	End (bp)	Size (bp)
3	50 039 303	51 137 089	1 097 786
3	88 327 613	90 194 622	1 867 009
3	155 929 451	165 555 125	9 625 674

Start and End refer to the positions in chromosome 3, hg19.

By exome sequencing, a total of 5419 variants (including single nucleotide variants, insertions and deletions) were found to be shared by the two affected siblings and absent in the unaffected one. Under a recessive model and based on the rarity of the phenotype presented by the family, we applied a series of filtering steps (Table 5) to select homozygous, autosomal, non‐synonymous variants that had not been previously reported at high minor allele frequencies in established databases containing exome sequencing data (dbSNP132, EVS or 1000 Genomes Project) and in our own HEX database containing exome sequencing data for neuropathologically normal elderly individuals.

**Table 5:** Bioinformatics analysis of whole‐exome sequencing in the studied family

	Individuals		
Filter step	II.1	II.2	II.3
Variants in coding regions or at splice sites	18 205	18 044	17 220
*and* homozygous	7832	7788	8140
*and* not in dbSNP 132, 1000Genomes or control exomes	24	23	25
*and* changes aminoacid or splice site	20	16	16
*and* shared between affected and absent from unaffected siblings	4		
*and* located within shared homozygosity regions	1 (*PTH1R*:p.Arg186His)		

Filtering pipeline applied to the studied family whole‐exome sequencing data.

**Figure 3:**
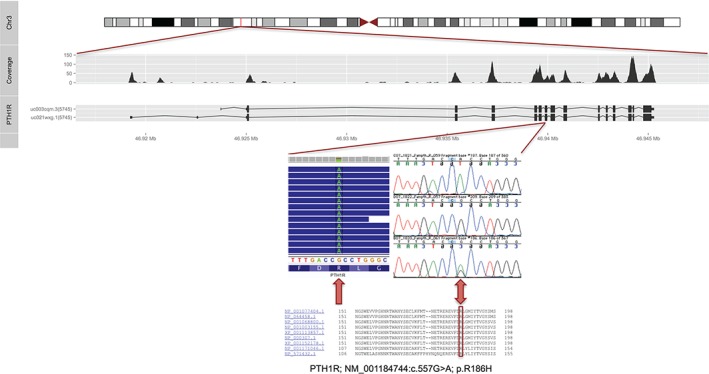
**Identification of the homozygous p.Arg186His mutation in PTH1R.** The top panel shows the region on chromosome 3 where PTH1R is located, with the coverage obtained from exome sequencing for each exon of the gene and both transcripts represented. The medium panel represents the variant calling obtained from exome sequencing at the p.Arg186His locus for one affected sibling, on the left, and Sanger sequencing traces for one affected and two unaffected samples from the family, on the right. The first sequence on the top corresponds to an affected individual (homozygous ‘AA’ at the p.Arg186His position), while the two bottom sequences show unaffected individuals (one homozygous wild‐type ‘GG’ and one heterozygous ‘GA’). The bottom panel shows the protein sequence comparison displaying high conservation across species: NP_0011077404.1: M. musculus; NP_064458.1: R. norvegicus; NP_001068800.1: B. taurus; NP_001003155.1: C. lupus; XP_001113857.1: M. mulatta; NP_000307.1: H. sapiens; XP_001152178.1: P. troglodytes; NP_001171046.1: G. gallus; NP_571432.1: D. rerio.

Only one novel variant was found after applying all filters and this was the *PTH1R* p.Arg186His, c.557G > A homozygous mutation. This variant is not present in the ExAC database, is highly conserved between species (Fig. 3), and is predicted by Polyphen‐2 and PROVEAN as probably damaging and deleterious. To confirm that we had not missed variants in genes within the homozygous regions, we calculated per‐base coverage for each sample at the segregating loci (see Supporting information).

Sanger sequencing was performed to confirm the mutation and to establish if it segregated with the disease in the studied family. Complete segregation was found, with all affected siblings being homozygous for the mutation and unaffected individuals being either heterozygous carriers or harbouring two reference alleles (Figs. 1, 3).

## Discussion

Initially, the proband's laboratorial presentation with severe hypocalcemia, hyperphosphatemia and normal levels of PTH was compatible with the diagnosis of primary HP. Later on, when she developed cognitive disturbance and PTH levels were slightly elevated, we were compelled to further investigate a PTH resistance syndrome. A PTH stimulation test was performed using subcutaneous recombinant human PTH‐(1–34). The results of PTH infusion test in our patient were remarkable for a blunted urinary cAMP, which is typical for PTH resistance syndromes. The normal phosphaturic response observed may be considered atypical in this context, but a similar dissociated response has been described previously in patients with PHP (Kharb *et al.* 2011; Lewin *et al.* 1978; O'Neill *et al.* 1981; Stogmann & Fischer 1975). Some explanations have been proposed, perhaps the more plausible one is that vitamin D supplementation normalizes the phosphaturic response (Kharb *et al.* 2011; Stogmann & Fischer 1975). An alternative hypothesis is that phosphaturic response to PTH may be independent of tubular adenylate cyclase stimulation in these patients (Stogmann & Fischer 1975). The integration of these results, with the high levels of PTH observed in other family members led us to propose the diagnosis of PHP. The further classification of PHP type 1b was based in the absence of bone malformation (osteodystrophy), as well as other endocrine defects, including normal thyroid function. We do not have an explanation for the unexpected normal PTH levels observed in our index case, which are divergent from the other two affected siblings and were possibly misleading. We can speculate whether this inability to increase PTH levels in response to a peripheral resistance is related to the severity of disease and especially to the inexorable evolution to dementia and akinetic mutism. In fact, PTH levels seem to modulate the severity of disease in all affected family members: subject II.4 has very high levels of PTH (365–500 pg/ml) and is asymptomatic; subject II.2 presents high PTH levels (172–198 pg/ml) and a mild phenotype (late onset cataracts and memory complaints); subject II.1 has mostly normal PTH levels and a severe cognitive deterioration. One other possible explanation, although highly unlikely, is the presence of two rare but very similar diseases in the same family. The unavailability of molecular data for *GNAS* in the affected siblings of the index case precludes the exclusion of this possibility (more details in Supporting Information).

### Neurological involvement

The phenomenology and severity of the neurological involvement deserves some further comment. Delirium and cognitive decline are manifestations of HP and PHP, being usually associated and maybe pathophysiologically related to the presence of intracranial calcification (Adorni *et al.* 2005; Baptista *et al.* 1997; El Otmani *et al.* 2013; Kowdley *et al.* 1999; Nicolai & Lazzarino 1994; Roca *et al.* 1995). Hypocalcemia seems to be a key factor for calcifications as well as for common neurological symptoms like tetany, muscle cramping and seizures, which usually respond quickly to calcium replacement (Friedman *et al.* 1987; Fujita 2004). This was the case for our patient, where neuromuscular hyperexcitability and seizures were easily controlled and remained stable during disease progression. Cognitive deficits may also constitute transitory symptoms of acute hypocalcemia, usually integrating a more complex state of confusion and delirium, during episodes of status epilepticus or as a manifestation of encephalopathy with reversible brain edema (Gupta *et al.* 2011; Hossain 1970; Palmer *et al.* 1959). The profile of cognitive impairment initially presented by our patient also configures the definition of a confusional sate or delirium related to metabolic encephalopathy (DSM IV; Association 1994). Hypocalcemia seems improbable according to the favourable evolution of other hypocalcemia‐related symptoms and the apparent dissociation between symptoms and calcium/phosphate serum levels (Table 1). Moreover, the patient had no clinical or imagiological signs of brain edema, and epilepsy was controlled. There is at least one report of a reversible dementia‐HP and consistent normocalcaemia with a rapid improvement and normalization of symptoms after therapy with 1,25‐dihydroxy‐cholecalciferol, suggesting that low levels of vitamin D could be a key factor (Stuerenburg *et al.* 1996). However, our patient was on a stable dose of calcitriol (0.25 µg/day) since the beginning of symptoms, indicating that this is not a likely explanation in this case.

A review of the literature revealed that stable and severe cognitive deficits and dementia have been essentially associated to chronic HP and PHP and widespread intracranial calcifications (Baptista *et al.* 1997; Cartier *et al.* 2002; El Otmani *et al.* 2013; Modrego *et al.* 2005). Dementia appears late in the course of the disease in most cases, and it is characterized as a subcortical dementia, probably secondary to mineral deposits in subcortical structures, leading to disruption of frontostriatal circuits and dysfunction of inter‐hemispheric relations (Cartier *et al.* 2002). Additionally, dementia, as well as hypokinetic rigidity, are considered late‐stage manifestations of PH, and the least likely symptoms to respond to therapy (Friedman *et al.* 1987). Our patient also configures this type of sub‐cortical frontal dementia, but with a more rapid and aggressive profile, and is paradigmatic of these overlapping syndromes, which may also indicate the existence of common pathological mechanisms subjacent to delirium and dementia. The mechanism of calcium deposition is not clear, but according to others may be due to chronic abnormalities of intra‐ and extracellular concentrations of calcium and phosphate (Fujita 2004; Goswami *et al.* 2012). Proposed mechanisms of extrapyramidal dysfunction and maybe dementia include altered vessel permeability to calcium and tissue ischaemia, abnormal synaptic excitability and ephaptic transmission, or local shifts in calcium concentration, possibly mediated by altered activity of the cerebral isoform of PTH responsive adenyl cyclase (Warren *et al.* 2002). In our opinion there were no other co‐morbidities in our patient to explain this dementia end‐stage. In fact, all forms of treatable dementia were excluded and there were no biomarkers of degenerative dementia, namely Alzheimer's disease. Besides, the observed clinical profile, with a fluctuating encephalopathy rapidly evolving to dementia with extrapyramidal features and finally to hypokinetic rigidity and mutism is atypical for degenerative dementias. The other affected family members are less symptomatic, but the clinical phenomenology of her sister is similar, presenting essentially with cognitive symptoms and extensive brain calcifications.

### PTH1R mutations

Mutations in *PTH1R* have previously been associated with five different diseases, reflecting the wide range of functions and pathways in which this receptor is involved. Homozygous mutations have been associated with Blomstrand's chondrodysplasia, a human embryonic lethal disorder characterized by advanced endochondral bone maturation, and with Eiken syndrome, presenting with skeletal features contrasting to the Blomstrand's phenotype and mainly characterized by retarded ossification (Duchatelet *et al.* 2005). Heterozygous mutations have been associated with Primary failure tooth eruption and with Jansen's metaphyseal chondrodysplasia (a rare form of dwarfism associated with hypercalcemia) (Decker *et al.* 2008; Schipani *et al.* 1995a). Additionally, germline and somatic mutations have been associated with Ollier disease (a type of enchondromatosis) (Hopyan *et al.* 2002), although this association is still to be confirmed (Rozeman *et al.* 2004). Table S2 summarizes the different types of mutations previously associated with these diseases. Here, we associate a novel homozygous missense mutation in *PTH1R* with PHP1b. The mutation, located in residue 186 of the protein, is predicted to be pathogenic by different prediction software and this residue, located just at the N‐terminal extracellular/transmembrane‐1 junction, has previously been shown to be essential for contact with position 13 of PTH (Adams *et al.* 1998). The proband in this family presents no symptoms of either Blomstrand's chondrodysplasia or Eiken syndrome and no family members reported delayed tooth eruption. This indicates that the location of the change in the protein is probably relevant for the downstream associated phenotype. The interaction between PTH and PTH1R has been predicted as a two‐site general interaction model involving two main components: (1) an interaction between the C‐terminal domain of the ligand and the N‐terminal domain of the receptor—mainly contributing to binding affinity; and (2) an interaction between the N‐terminal portion of the ligand and the juxtamembrane region of the receptor—contributing to signalling (Gardella & Juppner 2001). More specifically, point mutations at the neighbouring hydrophobic residues Phe184, Leu187 and Ile190 were shown to impair the interaction with PTH(3–34) and PTH(1–14) with a PTH(1–34) analog possibly crosslinking to PTH1R at Arg186 (Carter *et al.* 1999). The late onset of disease in this family is in accordance with the conclusion that the effect of two different mutations (p.Arg186Lys and p.Arg186Ala) engineered in the same residue as the mutation here described, is local and does not involve global conformational changes of the receptor. As such, this novel mutation (p.Arg186His) is expected to diminish the binding affinity and ligand specificity of the receptor, rather than abolish the overall function of PTH1R (Adams *et al.* 1998). In fact, a very similar phenotype to the one observed in this family has recently been reported to be associated with a novel homozygous mutation in *PTH*. The five previously known *PTH* mutations were all located in exon 2 of the gene that encodes the pre‐pro leader sequence of the hormone. All these mutations lead to inadequate secretion of the mature PTH(1–84) polypeptide. The novel PTH mutation (p.Arg25Cys) identified by Lee et al. is the first located within the α‐helical portion of PTH that mediates critical binding interactions with PTH1R and was shown to impair the PTH/PTH1R activation (Lee *et al.* 2015). The mutation we identified in *PTH1R* is expected to have a similar effect in this ligand‐receptor complex, possibly defining a novel form of PHP1b resulting from the impaired interaction between PTH and PTH1R.

In summary, the index case reported here represents the extreme end of the spectrum of cognitive impairment in PTH dysfunction and is a dramatic example of refractivity to treatment. Considering that this is also the first family identified to have PHP1b due to a mutation in *PTH1R*, we can speculate that this cognitive profile is the phenotype of this mutation. Furthermore, the resistance to PTH may be explained by an abnormal interaction between PTH and the PTH1R receptor, eventually modulated by a compensatory‐elevation of PTH. These are potentially interesting mechanisms to explore further with functional analyses.

## Supporting information


**Appendix S1:** Methods.

**Figure S1:** Homozygosity analysis. The left panel represents the large tracts of homozygosity shared between affected and absent in unaffected siblings across the entire genome and depicted as blue bars over the corresponding chromosome. Only three regions >1 Mb segregate with the disease in this family. The right panel shows the results for chromosome 3 from whole genome genotyping represented by the log ratio in the bottom and B allele frequencies for each of the six siblings. The pink vertical line indicates the location of PTH1R in chromosome 3. All affected siblings have large homozygous regions encompassing PTH1R while unaffected siblings show heterozygosity in the same locus.

**Figure S2:** Bone X‐ray of the proband. Bone X‐ray showing generalized osteopenia, but without skeletal abnormalities suggestive of Albright's hereditary osteodystrophy, Blomstrand's chondrodysplasia, Eiken skeletal dysplasia or Murk Jansen type of metaphyseal chondrodysplasia.

**Figure S3:** Cerebral SPECT of the index case. Cerebral SPECT disclosed cerebral hypoperfusion mainly at the frontal regions and basal ganglia, predominantly on the left side.
