# Supplementary material for: Pseudohypoparathyroidism type I‐b with neurological involvement is associated with a homozygous PTH1R mutation
Source: Genes Brain Behav. 2016 Aug 24;15(7):669–77. doi: 10.1111/gbb.12308 (PMC5026059; doi:10.1111/gbb.12308)
Supplement: Supplementary file 1 — Appendix S1: Methods. [file GBB-15-669-s004.docx]

# Supplementary Material

**Pseudohypoparathyroidism Type I-b with neurological involvement is associated with a homozygous PTH1R mutation**

Rita Guerreiro, José Brás, Sónia Batista, Paula Pires, Helena Ribeiro, Rosário Almeida, Catarina Oliveira, John Hardy, Isabel Santana

### METHODS

### Genetic analyses

## Exclusion of molecular changes in *GNAS*

The exclusion of molecular changes in *GNAS* was performed by direct sequencing, MLPA and methylation tests in the index case as a clinical diagnosis test. These analyses are not performed in our research laboratory and the additional analyses of *GNAS* in the siblings would have to be performed in a clinical setting. The informed consents obtained from these participants in the study were only associated with research analyses and no clinical diagnoses tests could be performed. Even though it is highly unlikely that two rare but very similar diseases would be present in the same family, this is still a possibility that we could not exclude due to the unavailability of *GNAS* molecular data on the siblings of the index case studied here.

## Exome sequencing

After exclusion of mutations in *GNAS* by direct sequencing, exome sequencing was performed in three individuals (II.1, II.2 and II.3, **Fig. 1**). The approach used for exome sequencing data analysis assumed a recessive mode of inheritance where shared variants could be determined in the affected cases and subsequently filtered out in the unaffected sibling. Genomic DNA was prepared according to Illumina’sTruSeq Sample Preparation v3 (Illumina, San Diego, CA, USA) and whole exome capture was performed with Illumina’sTruSeqExome Enrichment according to the manufacturer’s instructions. Sequencing was performed in Illumina’s HiSeq2000 using 100 bp paired-end reads. Following quality-control procedures, samples yielded between 5.6 and 9.9 Gb of high quality, aligned data. This amount of data represented mean target coverage between 35.1 and 67.6x; percentages of targets covered at greater than or equal to 10x of 86-91% and less than 0.5% of targets not being covered at least once. Sequence alignment and variant calling were performed against the reference human genome (hg19) using bwa (Li & Durbin, 2009) and the Genome Analysis Toolkit (McKenna *et al.*, 2010). PCR duplicates were removed prior to variant calling using the Picard software (<http://picard.sourceforge.net/index.shtml>). Based on the hypothesis that the mutation underlying this rare familial disease was not present in the general population, we excluded all common SNPs (MAF >0.01) identified in the 1000 Genomes project ([www.1000genomes.org/](http://www.1000genomes.org/)) or in dbSNP (<http://www.ncbi.nlm.nih.gov/projects/SNP/>Build 132).

## Sanger sequencing

ExonPrimer (ihg.gsf.de/ihg/ExonPrimer.html) was used to generate primers for amplification of *PTH1R* exon 6 plus exon-intron boundaries. The exon was PCR amplified using Roche FastStart PCR Master Mix polymerase (Roche Diagnostics, Risch-Rotkreuz, Switzerland). The PCR products were sequenced using the same forward and reverse primers with Applied Biosystems BigDye terminator v3.1 sequencing chemistry and run on an ABI3730xl genetic analyzer as per manufacturer’s instructions (Applied Biosystems, Foster City, CA, USA). The sequences were analyzed using Sequencher software, version 4.2 (Gene Codes, Ann Arbor, MI, USA) and mutations were named based on sequences with accession numbers NM_001184744.1 and NP_001171673.1. In silico analyses using PolyPhen-2 and PROVEAN were performed to predict mutations’ effects.

### RESULTS

### Detailed clinical presentation of the index case

At age 49, when complex partial seizures became recurrent, laboratory analysis revealed serum biochemical results relevant for a severe hypocalcemia (3.9mg/dl; reference value 8.1–10.4) and hyperphosphatemia (6.7 mg/dl; 3.0–5.0) with normal blood levels of albumin (4.1 g/dl; 3.5–5.6). Renal function and serum proteins were normal, serum magnesium was slightly decreased (1.5 mg/dl; 1.7–2.8) and a twenty four-hour calciuria evaluation was normal (87mg/24h; 50-300). Parathyroid hormone levels were normal (45pg/ml; 9-72).

At age 66, following an acute confusional state, comprehensive neurological and cognitive evaluations were performed.

The neurological examination revealed moderate to severe cognitive deterioration (see supplemental material for details) axial ataxia with no other cerebellar signs, paratonia and frontal release signs, slight rigidity of extremities and sporadic myoclonic jerks. There was no papilledema or evidence of neuromuscular hyperexcitability. Mental assessment disclosed emotional lability, psychomotor slowing, confabulation, echolalia, perseveration and attention deficit with fluctuation. Mental-State Examination-MMSE (Portuguese version) (Folstein *et al.*, 1975; Guerreiro M, 2003b) was abnormal (13/30) and the global score (49) on the Alzheimer’s disease Assessment Scale- ADAS-COG (Portuguese version) (Guerreiro M, 2003a; Mohs *et al.*, 1983; Rosen *et al.*, 1984) was compatible with moderate to severe cognitive deterioration.

Cognitive evaluation with the Battery of Lisbon for the Assessment of Dementia (BLAD) (Garcia, 1984; Guerreiro, 1998), which includes multiple neuropsychological tests representing key cognitive domains and is validated for the Portuguese population. This evaluation confirmed the observed attention disturbance (Digit span and cancelation tests) as well as short-term and episodic memory deficits (Wechsler Memory Scale). There was also marked frontal dysfunction with impairment in tests of motor control (Luria), verbal initiative (Verbal Semantic Fluency), verbal and non-verbal abstraction (Interpretation of Proverbs and the Raven Progressive Matrices) and executive/visuo-constructional abilities (Clock Drawing test). Language evaluation as well as tests related to social-autobiography knowledge was mildly impaired, although with fluctuating performance due to attention impairment and perseveration errors. This cognitive evaluation was compatible with a progressive encephalopathy and/or dementia probably related to calcium metabolism disturbance. Routine laboratorial tests for treatable dementia were normal/negative (including chemistry profile, CBC count, thyroid function tests, vitamin B12 and folic acid level, syphilis and Lyme serology). ECG was normal and EEG showed frontal intermittent rhythmic delta activity (FIRDA) and bilateral temporal paroxysmal activity. Radiological investigations showed generalized osteopenia without typical skeletal abnormalities suggestive of Albright’s hereditary osteodystrophy, Blomstrand’s chondrodysplasia, Eiken skeletal dysplasia or Murk Jansen type of metaphyseal chondrodysplasia (**Fig. S2**). Brain CT scan revealed extensive calcifications of the basal ganglia, thalamus, periventricular white matter, centrum semiovale and at the dentate nuclei and cerebellar white matter (**Fig. 2**). Cerebral SPECT disclosed cerebral hypoperfusion mainly at the frontal regions and basal ganglia, predominantly on the left side (**Fig. S3**). CSF analysis was normal, including the levels of Aβ-42 (1151.8 pg/mL; reference values >500 pg/mL), tau (241.5 pg/mL; reference values <276 pg/mL) and p-tau (25,1 pg/mL; reference values <61pg/ml), excluding the improbable diagnosis of Alzheimer’s disease. Over the next two years the patient developed progressive motor and cognitive decline with apathy, stereotyped behavior, echolalia and nocturnal agitation.

### Exome sequencing analysis

In order to assess the coverage within the three extended homozygous regions identified as segregating with disease in the studied family, we calculated per base coverage of each exonic sequence for genes located within these regions (**Table S1**). There are a total of 151 genes in these loci and only 3 had an average coverage below 33x (*LOC100132146*, *C3orf80* and *SCHIP1*). Although we cannot exclude these three genes as potentially carrying disease-causing mutations, these data show that we have achieved adequate coverage depth of the coding homozygous regions.

**Table S1.** **Average of per base coverage within the segregating homozygosity regions**

| Gene | Average coverage |
| --- | --- |
| *LOC100132146* | 0.14 |
| *C3orf80* | 0.88 |
| *SCHIP1* | 12.28 |
| *TMEM212* | 33.77 |
| *CSPG5* | 38.25 |
| *HYAL1* | 38.5 |
| *CAMP* | 43 |
| *TMEM158* | 45.76 |
| *CCDC12* | 46.33 |
| *HYAL2* | 47.73 |
| *PRSS50* | 50.3 |
| *CDCP1* | 50.34 |
| *LRRC34* | 50.84 |
| *VEPH1* | 51.11 |
| *SEMA3B* | 53.2 |
| *TREX1* | 55.47 |
| *LEKR1* | 55.84 |
| *MYNN* | 56.74 |
| *NAT6* | 57.05 |
| *LTF* | 57.39 |
| *ISL2* | 57.39 |
| *SEMA3F* | 57.83 |
| *PLXNB1* | 58.21 |
| *C3orf55* | 58.22 |
| *TMEM115* | 59.24 |
| *PTPN23* | 60.41 |
| *ALS2CL* | 60.74 |
| *SCAP* | 61 |
| *SHISA5* | 61.3 |
| *HYAL3* | 62.91 |
| *SLC6A20* | 62.99 |
| *NBEAL2* | 63.01 |
| *GNAT1* | 63.59 |
| *KIF9* | 63.83 |
| *CCDC51* | 64.33 |
| *ZBBX* | 64.43 |
| *UCN2* | 65.21 |
| *IQCJ* | 65.34 |
| *NME6* | 65.55 |
| *CISH* | 65.67 |
| *DHX30* | 66.29 |
| *GNAI2* | 67.01 |
| *B3GALNT1* | 67.77 |
| *PSTPIP1* | 68.36 |
| *RASSF1* | 68.52 |
| *EPHA6* | 68.73 |
| *TUSC2* | 68.87 |
| *C15orf27* | 69.66 |
| *LXN* | 69.7 |
| *RARRES1* | 69.71 |
| *XCR1* | 70.1 |
| *PTH1R* | 70.29 |
| *GBE1* | 70.32 |
| *SERPINI1* | 70.47 |
| *IQCJ-SCHIP1* | 70.55 |
| *RTP3* | 71.03 |
| *PRSS42* | 71.32 |
| *TIPARP* | 71.56 |
| *SI* | 72.5 |
| *ATRIP* | 72.6 |
| *WDR49* | 72.97 |
| *PDCD10* | 73.25 |
| *KPNA4* | 73.54 |
| *IFRD2* | 73.68 |
| *IFT80* | 73.9 |
| *CYB561D2* | 73.91 |
| *CLDN11* | 74.21 |
| *C3orf18* | 74.7 |
| *RPL22L1* | 75.18 |
| *GOLIM4* | 75.87 |
| *CACNA2D2* | 76.5 |
| *SERPINI2* | 76.61 |
| *PFKFB4* | 77.03 |
| *OTOL1* | 77.3 |
| *IL12A* | 77.5 |
| *MAPKAPK3* | 77.67 |
| *ZMYND10* | 77.84 |
| *C3orf45* | 78.12 |
| *FNDC3B* | 78.28 |
| *SHOX2* | 78.91 |
| *LRRC2* | 79.16 |
| *SCAPER* | 80.49 |
| *LRP1B* | 80.72 |
| *UNKNOWN* | 81.37 |
| *SSR3* | 81.59 |
| *NPRL2* | 81.91 |
| *SEC62* | 82.46 |
| *TNIK* | 83.9 |
| *ARL14* | 84.05 |
| *HEMK1* | 84.13 |
| *LIMD1* | 84.18 |
| *MLF1* | 84.33 |
| *C3orf75* | 84.83 |
| *EIF5A2* | 85.5 |
| *PRSS45* | 85.98 |
| *TRIM59* | 86.1 |
| *MFSD1* | 86.16 |
| *CDC25A* | 86.32 |
| *GPR160* | 86.36 |
| *PTX3* | 86.67 |
| *PLD1* | 87.05 |
| *BCHE* | 87.15 |
| *RCN2* | 87.2 |
| *SLC38A3* | 87.34 |
| *LZTFL1* | 88.28 |
| *PHC3* | 88.48 |
| *MECOM* | 89.24 |
| *SAMD7* | 89.27 |
| *SLC7A14* | 89.87 |
| *LARS2* | 90.55 |
| *CCRL2* | 90.77 |
| *PRKCI* | 91.06 |
| *KCNAB1* | 91.28 |
| *MYL3* | 91.59 |
| *SMARCC1* | 92.07 |
| *RBM6* | 92.35 |
| *MAP4* | 92.81 |
| *SPTSSB* | 93.1 |
| *ETFA* | 93.47 |
| *SMC4* | 94.55 |
| *SLITRK3* | 95.24 |
| *DOCK3* | 95.55 |
| *SLC2A2* | 95.69 |
| *RSRC1* | 95.76 |
| *NMD3* | 96.46 |
| *EPHA3* | 96.57 |
| *RBM5* | 97.19 |
| *TMIE* | 97.32 |
| *CCNL1* | 97.57 |
| *LRRC31* | 98.07 |
| *FBXW12* | 98.28 |
| *CCR1* | 98.75 |
| *SACM1L* | 98.92 |
| *FYCO1* | 100.15 |
| *SKIL* | 100.36 |
| *COL7A1* | 100.75 |
| *KLHL18* | 104.72 |
| *SPINK8* | 106.15 |
| *GFM1* | 107.05 |
| *SETD2* | 112.79 |
| *ARPM1* | 117.17 |
| *LRRIQ4* | 117.54 |
| *CCR9* | 118.47 |
| *PPM1L* | 122.83 |
| *CCR5* | 131.77 |
| *CCR3* | 138.33 |
| *CXCR6* | 142.73 |
| *CCDC72* | 145.54 |
| *CCR2* | 169.62 |
| *TDGF1* | 193.38 |
| *ZNF589* | 199.59 |

### DISCUSSION

**Table S2. Types of mutations previously identified in PTH1R and correspondence with the different phenotypes**

| Disease | Mutations zygosity | Types of mutation identified |
| --- | --- | --- |
| Blomstrand’s chondrodysplasia | Homozygosity and compound heterozygosity | Missense, intronic deletions and transitions affecting splice sites, nonsense |
| Eiken syndrome | Homozygosity | Nonsense |
| Primary failure tooth eruption | Heterozygosity | Intronic transition and transversions affecting splice site |
| Jansen’s metaphyseal chondrodysplasia | Heterozygosity | Missense |
| Ollier disease | Heterozygosity | Germline and somatic mutations |

Different types of mutations and diseases as described in OMIM.

### SUPPLEMENTARY FIGURES LEGENDS

## Figure S1. Homozygosity analysis.

The left panel represents the large tracts of homozygosity shared between affected and absent in unaffected siblings across the entire genome and depicted as blue bars over the corresponding chromosome. Only three regions >1Mb segregated with the disease in this family. The right panel shows the results for chromosome 3 from whole genome genotyping represented by the log ratio in the bottom and B allele frequencies for each of the six siblings. The pink vertical line indicates the location of *PTH1R* in chromosome 3. All affected siblings have large homozygous regions encompassing *PTH1R* while unaffected siblings show heterozygosity in the same locus.

## Figure S2. Bone X-Ray of the proband.

Bone X-Ray showing generalized osteopenia, but without skeletal abnormalities suggestive of Albright’s hereditary osteodystrophy, Blomstrand’s chondrodysplasia, Eiken skeletal dysplasia or Murk Jansen type of metaphyseal chondrodysplasia.

## Figure S3. Cerebral SPECT of the index case.

Cerebral SPECT disclosed cerebral hypoperfusion mainly at the frontal regions and basal ganglia, predominantly on the left side.

### REFERENCES

Folstein, M.F., Folstein, S.E. & Mchugh, P.R. (1975) "Mini-mental state". A practical method for grading the cognitive state of patients for the clinician. *J Psychiatr Res* **12,** 189-198.

Garcia, C. 1984. *A Doença de Alzheimer: Problemas de diagnóstico clínico.* PhD PhD Dissertation, Faculdade de Medicina de Lisboa.

Guerreiro, M. 1998. *Contributo da Neuropsicologia para o estudo das Demências.* PhD PhD dissertation, Faculdade de Medicina de Lisboa.

Guerreiro M, F.S., Barreto J (2003a) Escalas e Testes na Demência. *Grupo de Estudos de Envelhecimento Cerebral e Demência***,** 33-49.

Guerreiro M, S.A., Botelho Ma (2003b) Avaliação Breve do Estado Mental: Escalas e Testes na Demência. *Grupo de Estudos de Envelhecimento Cerebral e Demência***,** 27-32.

Li, H. & Durbin, R. (2009) Fast and accurate short read alignment with Burrows-Wheeler transform. *Bioinformatics* **25,** 1754-1760.

Mckenna, A., Hanna, M., Banks, E., Sivachenko, A., Cibulskis, K., Kernytsky, A., Garimella, K., Altshuler, D., Gabriel, S., Daly, M. & Depristo, M.A. (2010) The Genome Analysis Toolkit: a MapReduce framework for analyzing next-generation DNA sequencing data. *Genome research* **20,** 1297-1303.

Mohs, R.C., Rosen, W.G. & Davis, K.L. (1983) The Alzheimer's disease assessment scale: an instrument for assessing treatment efficacy. *Psychopharmacol Bull* **19,** 448-450.

Rosen, W.G., Mohs, R.C. & Davis, K.L. (1984) A new rating scale for Alzheimer's disease. *Am J Psychiatry* **141,** 1356-1364.
