# Supplementary figures and images for: Pseudohypoparathyroidism type I‐b with neurological involvement is associated with a homozygous PTH1R mutation
Source: Genes Brain Behav. 2016 Aug 24;15(7):669–77. doi: 10.1111/gbb.12308 (PMC5026059; doi:10.1111/gbb.12308)

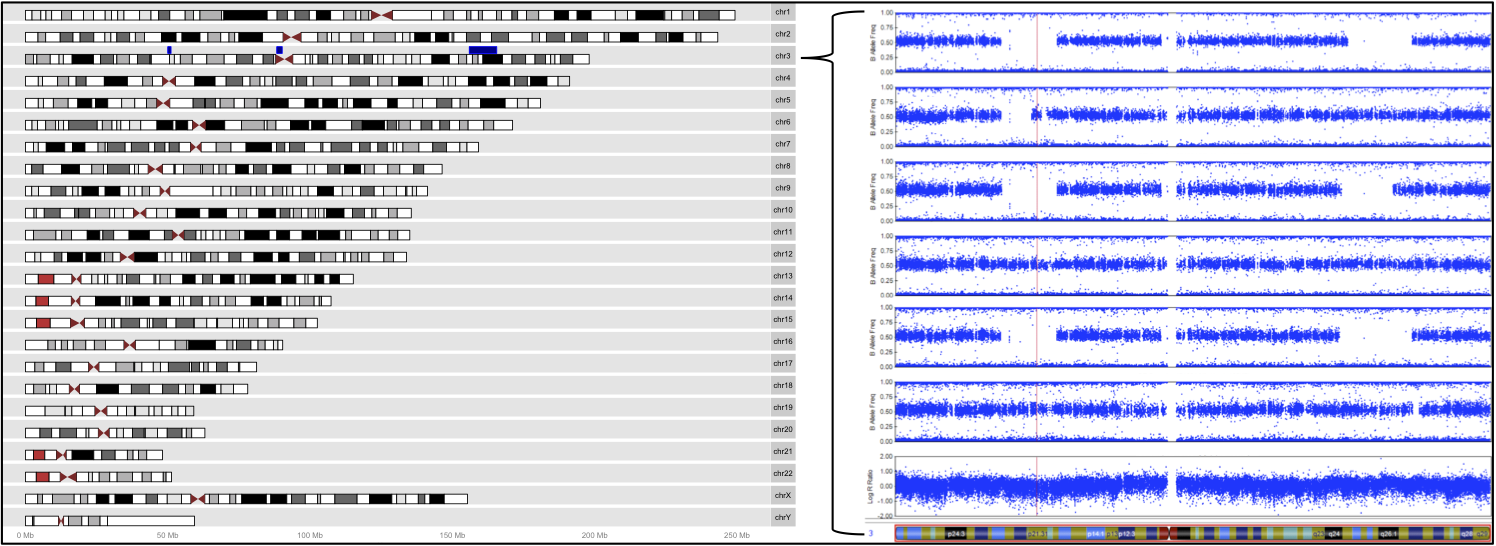

Supplement: Supplementary file 2 — Figure S1: Homozygosity analysis. The left panel represents the large tracts of homozygosity shared between affected and absent in unaffected siblings across the entire genome and depicted as blue bars over the corresponding chromosome. Only three regions >1 Mb segregate with the disease in this family. The right panel shows the results for chromosome 3 from whole genome genotyping represented by the log ratio in the bottom and B allele frequencies for each of the six siblings. The pink vertical line indicates the location of PTH1R in chromosome 3. All affected siblings have large homozygous regions encompassing PTH1R while unaffected siblings show heterozygosity in the same locus. [file GBB-15-669-s001.tiff]

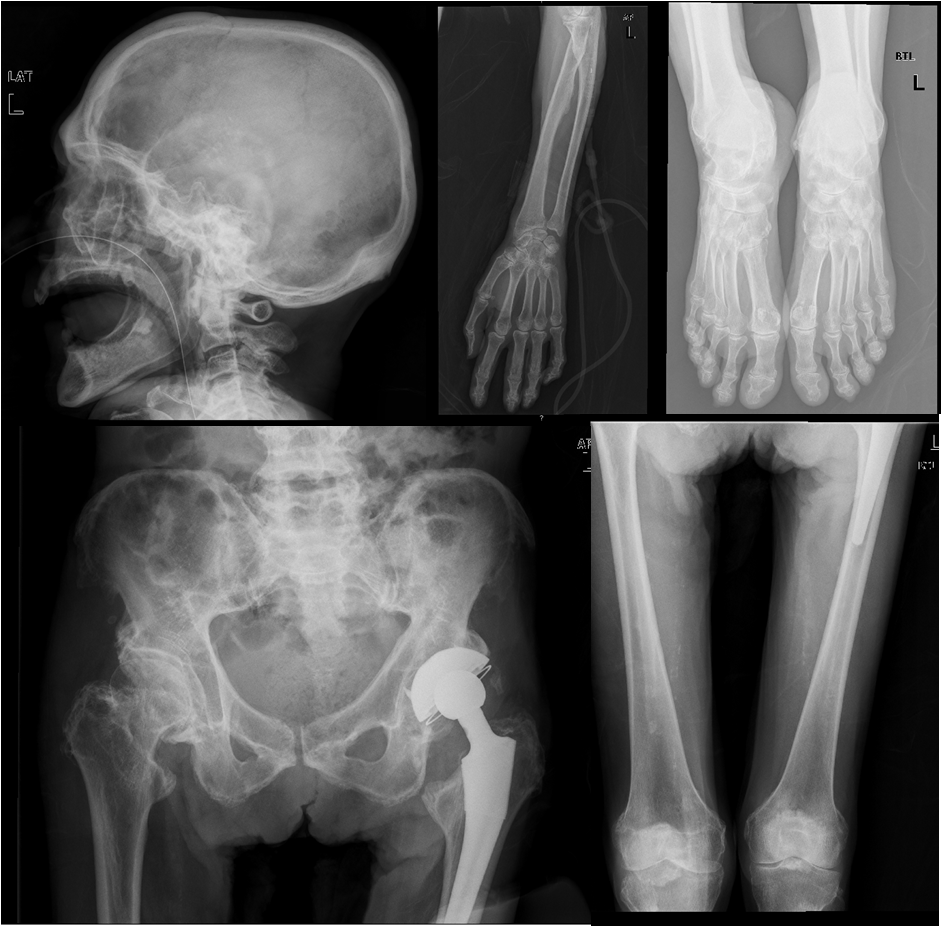

Supplement: Supplementary file 3 — Figure S2: Bone X‐ray of the proband. Bone X‐ray showing generalized osteopenia, but without skeletal abnormalities suggestive of Albright's hereditary osteodystrophy, Blomstrand's chondrodysplasia, Eiken skeletal dysplasia or Murk Jansen type of metaphyseal chondrodysplasia. [file GBB-15-669-s002.tiff]

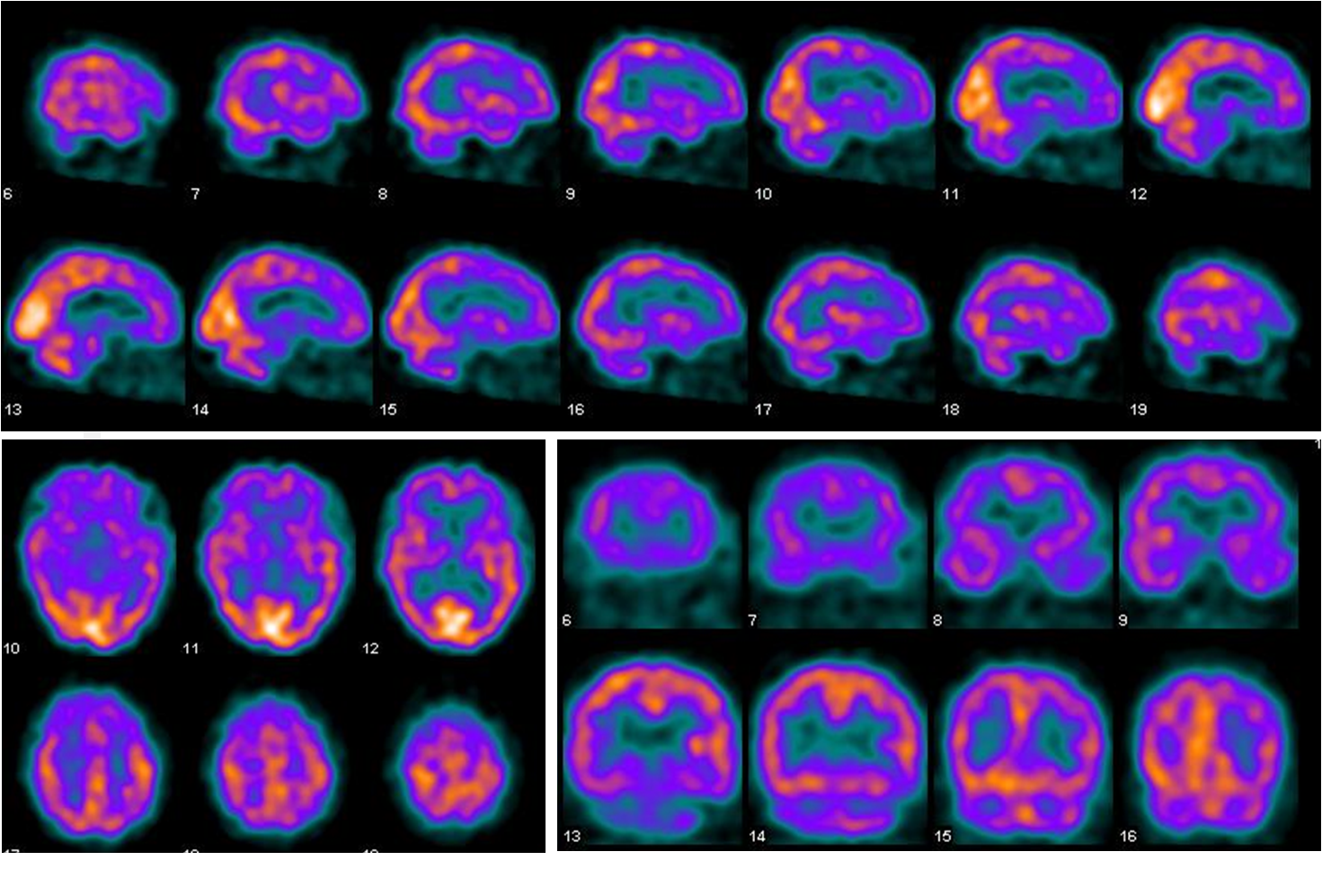

Supplement: Supplementary file 4 — Figure S3: Cerebral SPECT of the index case. Cerebral SPECT disclosed cerebral hypoperfusion mainly at the frontal regions and basal ganglia, predominantly on the left side. [file GBB-15-669-s003.tiff]
